# Synergism from combinations of infection-blocking malaria vaccines

**DOI:** 10.1186/1475-2875-12-280

**Published:** 2013-08-08

**Authors:** Michael T White, David L Smith

**Affiliations:** 1 Department of Infectious Disease Epidemiology, MRC Centre for Outbreak Analysis and Modelling, Imperial College, London, UK; 2 Department of Epidemiology, Johns Hopkins Bloomberg School of Public Health, Baltimore, Maryland, USA; 3 Fogarty International Center, National Institutes of Health, Baltimore, Maryland, USA

**Keywords:** Malaria, Vaccine, Infection, Pre-erythrocytic, Synergy

## Abstract

*Plasmodium falciparum* infections present novel challenges for vaccine development, including parasite replication dynamics not previously encountered for viral pathogens, and enormous diversity in target antigens. These challenges are illustrated by using a mathematical model to describe the association between the proportion of pre-erythrocytic or blood-stage parasites eliminated by vaccine-induced immune responses and the proportion of infections prevented. It is hypothesized that due to the requirement for all sporozoites to be eliminated to prevent infection, combining infection-blocking vaccines that confer protection through different biological mechanisms could lead to synergistic combinations of efficacy. Vaccines targeting blood-stage parasites may also combine synergistically if they combine to reduce the parasite multiplication rate to below the threshold of 1.

## Background

*Plasmodium falciparum* infections present novel challenges for vaccine development, including parasite replication dynamics not previously encountered for viruses, and enormous diversity in target antigens [1]. Malaria parasites express multiple life stages in humans, each presenting unique targets for vaccine-induced immune responses. Malaria infection can be prevented either by targeting the pre-erythrocytic (PE) stages and clearing sporozoites inoculated in the skin or infected hepatocytes in the liver, or by targeting blood-stage (BS) merozoites and infected red blood cells in the prepatent period after emergence from the liver and before parasite densities increase to detectable levels. The dynamics of these parasite life stages and corresponding immune kinetics also differ. Sporozoites pass from the skin to the liver without replicating, so clearing sporozoites or eliminating hepatocytes can prevent infection outright. The pass-through system also means that the immune system will be naturally exposed to and stimulated by relatively few sporozoites, but this number increases after repeated exposure to infectious bites of mosquitoes. BS parasites undergo periodic replication, so like viral pathogens, the combined vaccine-induced and naturally acquired immune response can prevent infection if enough parasites are cleared so that the number of parasites is reduced from one generation to the next. Replication of BS parasites implies that the immune system will be exposed to very high parasite numbers from a single infection.

The sporozoite bottleneck would seem to make an easy vaccine target, but the potential for a single parasite to evade vaccine-induced immune responses and spark an infection makes the challenge of developing an effective PE vaccine deceptively difficult. The effect of BS malaria vaccines on BS parasites is qualitatively similar to successful vaccines against viruses, in that a threshold immunological correlate for sterile protection against infection is theoretically possible. Hepatic parasites that survive the PE immune response will release merozoites into the blood stream which invade and replicate within red blood cells, causing an increase in parasite numbers every two-day cycle by a factor defined as the parasite multiplication rate (PMR) [2]. PMR >1 will lead to an increase in parasite numbers and possibly an episode of clinical malaria, and PMR <1 will lead to a decrease in parasite numbers and the clearance of BS infection. BS vaccines prevent the replication of BS parasites by either preventing merozoites from invading red blood cells or clearing infected red blood cells. A BS vaccine that fails to reduce PMR <1 may still confer protection against clinical and severe episodes by providing time for the natural immune response to mature. In a study in *Aotus* monkeys, a reduction in median PMR from 15 to 5 in the early phase of infection was associated with subsequent clearance of parasites without developing symptoms of anaemia (Sandy Douglas – personal communication). In contrast to BS vaccines, which need not clear every parasite, PE vaccines must prevent the successful development of each sporozoite, a challenging prospect given that most evidence suggests that the number of sporozoites inoculated per infectious bite is highly skewed, with some bites injecting very large numbers of sporozoites [3].

### The potential benefits of multicomponent malaria vaccines

**Figure 1:**
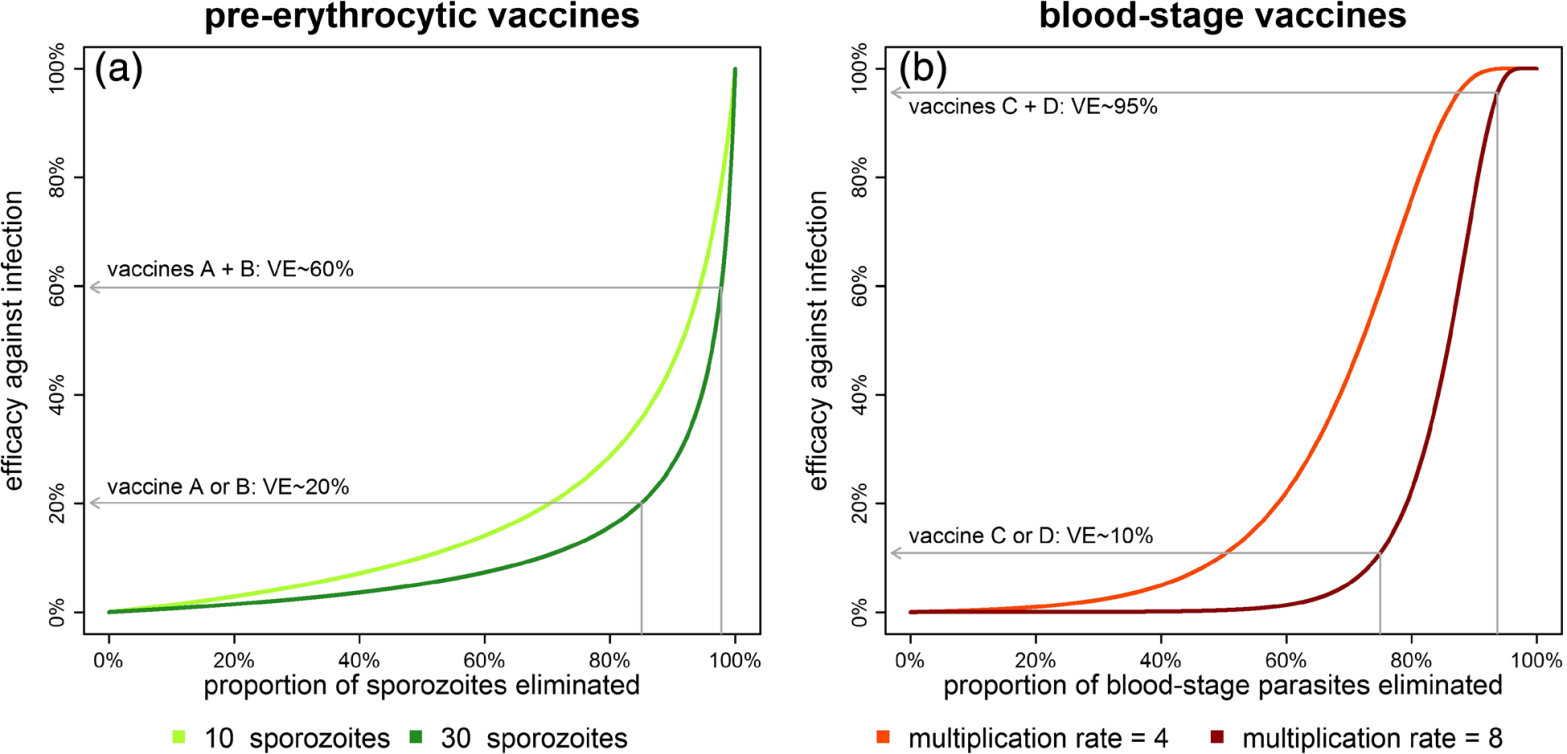
**Estimated efficacy against infection as a function of the average proportion of parasites killed for pre-erythrocytic (green) and blood-stage vaccines (red). (a)** The number of successfully developing sporozoites is assumed to follow a Negative Binomial distribution with mean *n* and shape parameter *r* = 0.5. With a mean of *n =* 30 sporozoites, a pre-erythrocytic vaccine that eliminates 85% of sporozoites will prevent 20% of infections. Combining two vaccines that eliminate 85% of sporozoites via independent mechanisms will result in a multicomponent vaccine that prevents 60% of infections. The curve is convex such that incremental increases in the proportion of sporozoites eliminated translate to greater increases in efficacy against infection. **(b)** Blood-stage parasites are assumed to increase in number every two-day cycle by a factor equal to the parasite multiplication rate (PMR), which varies among individuals according to a Log-Normal distribution with coefficient of variation *C*_*v*_ = 0.5. With a mean PMR = 8, a blood-stage vaccine that eliminates 75% of parasites will prevent only 10% of infections. Combining two vaccines that eliminate 75% of parasites through independent mechanisms will result in a multi-component vaccine preventing 95% of infections. The point of inflection in the curve corresponds to the proportion of BS parasites that must be eliminated to reduce the PMR below the threshold of 1.

Although both PE and BS vaccines induce immune responses that target individual parasites within the human host, the relationship between the average proportion of parasites killed and the proportion of infections prevented (efficacy against infection) is qualitatively different, as illustrated in Figure 1. Given that a single sporozoite evading the vaccine-induced immune response is sufficient to initiate malaria infection, and that during some mosquito bites large numbers of sporozoites may be inoculated, it is not predicted that there will be any threshold immunological correlate of protection at which sterile protection from infection is conferred, and hence that pre-erythrocytic malaria vaccines will be leaky [4]. This could explain, in part, why RTS,S has produced such high antibody responses, yet estimates of efficacy against infection are approximately 50% [5]. The convex shape of the curve for PE vaccines in Figure 1a suggests that preventing 50% of infections requires that, on average, in excess of 90% of sporozoites be killed [6]. The steepness of the curve in this region implies that incremental improvements in the proportion of parasites killed can lead to substantial improvements in efficacy against infection as the last few sporozoites are cleared up. An implication of this is that if two PE vaccines providing protection through different biological mechanisms are co-administered, efficacies are expected to combine synergistically. For example, if two vaccines kill 85% of sporozoites and prevent 20% of infections when administered on their own, they will kill 1 - (1–0.85)^2^ ≈ 98% of sporozoites when combined, but prevent approximately 60% of infections. There is also the potential for synergism for a combination of two BS vaccines, but this depends on whether the combined effect of the two vaccines is sufficient to reduce the PMR below the threshold of 1 in a large proportion of the vaccinated cohort. The threshold proportion of BS parasites killed to prevent infection corresponds to the inflection point of the curve in Figure 1b.

The challenge to vaccine developers in selecting an antigen to effectively target, may be turned into an opportunity to target multiple antigens [7] with efficacy combining synergistically. Synergy will depend not just on the initial efficacy after vaccination, but on the duration of each component vaccine, with waning driven by the vaccine with the shortest half-life.

### Combining existing vaccines may lead to synergistic improvements in efficacy

RTS,S has been shown to prevent approximately 50% of infections by eliminating >95% of sporozoites [8]. It is hypothesized that, due to the requirement for all sporozoites to be eliminated to prevent infection, combining RTS,S with a second vaccine that targets sporozoites via a different mechanism could lead to a multicomponent vaccine that prevents substantially more than 50% of infections. This would hold even if the second vaccine has negligible efficacy against infection when administered on its own, so long as it eliminates sufficient numbers of sporozoites [9], and there is no immunological interference between the two vaccines [10]. This phenomenon may also explain the high levels of efficacy against infection following immunization by irradiated sporozoites [11] which may confer protection by simultaneously inducing multiple, independent immune responses directed towards *P. falciparum* sporozoites.

## Competing interests

The authors declare that they have no competing interests.

## Authors’ contributions

MTW and DLS contribute to the writing of the manuscript. Both authors read and approved the final manuscript.
